# High-throughput optical action potential recordings in hiPSC-derived cardiomyocytes with a genetically encoded voltage indicator in the *AAVS1* locus

**DOI:** 10.3389/fcell.2022.1038867

**Published:** 2022-10-07

**Authors:** Fangfang Zhang, Anna B. Meier, Christine M. Poch, Qinghai Tian, Stefan Engelhardt, Daniel Sinnecker, Peter Lipp, Karl-Ludwig Laugwitz, Alessandra Moretti, Tatjana Dorn

**Affiliations:** ^1^ First Department of Medicine, Cardiology, Klinikum rechts der Isar, Technical University of Munich, School of Medicine and Health, Munich, Germany; ^2^ Molecular Cell Biology, Centre for Molecular Signaling (PZMS), Medical Faculty, Saarland University, Homburg, Germany; ^3^ Institute of Pharmacology and Toxicology, Technical University of Munich, Munich, Germany; ^4^ DZHK (German Centre for Cardiovascular Research), Partner Site Munich Heart Alliance, Munich, Germany

**Keywords:** voltage-sensitive fluorescent protein (VSFP), Förster resonance energy transfer (FRET), optical action potential (AP) recording, *AAVS1* safe harbor locus, hiPSC-derived cardiomyocytes, 3D culture

## Abstract

Cardiomyocytes (CMs) derived from human induced pluripotent stem cells (hiPSCs) represent an excellent *in vitro* model in cardiovascular research. Changes in their action potential (AP) dynamics convey information that is essential for disease modeling, drug screening and toxicity evaluation. High-throughput optical AP recordings utilizing intramolecular Förster resonance energy transfer (FRET) of the voltage-sensitive fluorescent protein (VSFP) have emerged as a substitute or complement to the resource-intensive patch clamp technique. Here, we functionally validated our recently generated voltage indicator hiPSC lines stably expressing CAG-promoter-driven VSFP in the *AAVS1* safe harbor locus. By combining subtype-specific cardiomyocyte differentiation protocols, we established optical AP recordings in ventricular, atrial, and nodal CMs in 2D monolayers using fluorescence microscopy. Moreover, we achieved high-throughput optical AP measurements in single hiPSC-derived CMs in a 3D context. Overall, this system greatly expands the spectrum of possibilities for high-throughput, non-invasive and long-term AP analyses in cardiovascular research and drug discovery.

## Introduction

In the past decade, human induced pluripotent stem cell-derived cardiomyocytes (hiPSC-CMs) have opened unprecedented possibilities to investigate mechanisms of human cardiovascular diseases (Moretti et al., 2010; Dorn et al., 2018; Bekhite and Schulze, 2021; Funakoshi and Yoshida, 2021; Krane et al., 2021; Micheu and Rosca, 2021; Meier et al., 2022) and to explore novel treatment options (Gramlich et al., 2015; Csobonyeiova et al., 2016; Gharanei et al., 2022). They have also served as a safety pharmacology platform to assess drug-induced cardiotoxicity, including life-threatening proarrhythmia, a major concern in pharmaceutical development (Gintant et al., 2017).

Across diverse applications, recording the action potentials (APs) of hiPSC-CMs is essential to detect changes in their electrophysiological function. Traditional cellular electrophysiology measurements are performed using patch-clamp techniques developed in the late 1970’s (Neher and Sakmann, 1976). Although this method remains the gold standard for the assessment of cellular electrical parameters, it is not suitable for high-throughput and long-term monitoring of APs of hiPSC-CMs due to its time-consuming, highly-invasive nature and difficulties in achieving recordings over extended time periods. Voltage-sensitive dyes represent an alternative approach for AP measurements allowing non-invasive optical imaging; however, they have limited potential for repeated long-term recordings due to phototoxicity (Hardy et al., 2009; Miller et al., 2012; Asakura et al., 2015; Kopljar et al., 2018). In contrast, genetically encoded voltage indicators (GEVIs), including the voltage-sensitive fluorescent protein (VSFP), do not have phototoxic effects and, thus, enable long-term studies of APs without the need for externally-added dyes, offering another strategy for AP measurements that was first used to investigate neuronal excitability (Akemann et al., 2009; Akemann et al., 2012; Lam et al., 2012). The VSFP reporter consists of a voltage-sensing transmembrane domain fused to a pair of fluorescent proteins Clover (GFP variant) and mRuby2 (RFP variant) (Lam et al., 2012). The optical detection of APs is based on changes in Förster resonance energy transfer (FRET) efficiency between two fluorescent reporters (Lam et al., 2012).

Using lentivirus and adeno-associated virus (AAV) constructs, VSFP-based voltage sensors have already been applied to faithfully report membrane potentials in hiPSC-CMs (Chen et al., 2017; Goedel et al., 2018; Esfandyari et al., 2022). Lentiviral vectors have the advantage of providing high expression of the transgenes, however, they might be toxic to the cells and can randomly integrate into the host genome leading to potentially undesirable consequences (Rothe et al., 2013; Schlimgen et al., 2016). In addition, transgenes delivered by lentiviral vectors to hiPSCs may become silenced during differentiation (Pfaff et al., 2013). AAVs do not integrate into the host genome compared to the lentiviruses (Chamberlain et al., 2017), however, both viral systems have been proven to inefficiently transduce CMs in 3D context.

In this study, we functionally characterized our recently generated stable hiPSC lines constitutively expressing VSFP under the CAG promoter inserted into the adeno-associated virus integration site 1 (*AAVS1*) safe harbor locus (AAVS1-VSFP-hiPSC) (Zhang et al., 2022), which can be used for assessment of electrophysiological properties of hiPSC-CMs. We further demonstrated that the expression of VSFP is maintained and even increased during *in vitro* cardiomyocyte differentiation. Taking advantage of subtype-specific CM differentiation protocols, we developed a convenient system for optical APs recordings in ventricular, atrial, and nodal cells. Importantly, in the context of cardiac tissue engineering, we could measure optical APs in single CMs in a 3D tissue environment, demonstrating the utility of genetically encoded VSFP in complex cardiac models with physiological relevance.

## Results

### Generation of high-performance voltage sensor hiPSC lines by insertion of a CAG-VSFP cassette into the *AAVS1* safe harbor locus

The voltage indicator applied here is the voltage-sensitive fluorescent protein variant VSFP-CR (Akemann et al*.*, 2009; Akemann et al*.*, 2012) (Supplementary Figure S1A). VSFP-CR consists of a voltage-sensitive transmembrane domain fused to a pair of green (Clover, GFP variant) and red (mRuby2, RFP variant) fluorescent proteins. Due to the close proximity of the two fluorophores, the excitation of the green fluorescent protein results in a fraction of the excitation energy being transferred to the red fluorescent protein *via* FRET, resulting in emission from the green and the red fluorescent proteins (Supplementary Figure S1A, left panel). Upon depolarization, a structural rearrangement of the voltage sensor triggers a reorientation of the two fluorescent proteins increasing the efficiency of FRET (Supplementary Figure S1A, right panel). APs are therefore detected as changes of the relative intensity of green and red fluorescence over time.

For the generation of the hiPSC lines expressing a genetically encoded VSFP (Zhang et al., 2022), we used CRISPR/Cas9 to target the adeno-associated virus integration site 1, namely, the intron 1 of the *PPP1R12C* gene on chromosome 19, encoding the protein phosphatase 1 (Oceguera-Yanez et al., 2016) (Supplementary Figure S1B). This region, known as a safe harbor site, allows robust and stable transgene expression and has been widely used to express reporters and other transgenes in embryonic and pluripotent stem cells (Smith et al., 2008; Ran et al., 2013a; Ran et al., 2013b; Hockemeyer and Jaenisch, 2016; Oceguera-Yanez et al., 2016). Moreover, disruption of this region through a transgene insertion does not have any adverse effects on the cell (Kotin et al., 1992). We chose the synthetic CAG promoter to drive the expression of the VSFP indicator, since it has been reported to promote robust gene expression in pluripotent stem cells and their differentiated derivatives (Bertero et al., 2016; Oceguera-Yanez et al*.*, 2016).

After clone selection and PCR screening of the targeted cells (Supplementary Figures S1C, S1D) we obtained 42% homozygous and 54% heterozygous clones (Supplementary Figure S1E). One homozygous and one heterozygous clone were selected for further validation and were registered in the Human Pluripotent Stem Cell Registry (heterozygous ID MRIi003-A-5; homozygous ID MRIi003-A-6) (Zhang et al., 2022). Both lines expressed Clover (GFP) and mRuby2 (RFP) and the intensity of the reporter signal was stronger in the homozygous compared to the heterozygous line, as detected by flow cytometry (Supplementary Figure S1F).

### Optical recordings of spontaneous and stimulated APs in AAVS1-VSFP-hiPSC-derived ventricular cardiomyocytes (AAVS1-VSFP-hiPSC-vCMs)

**FIGURE 1:**
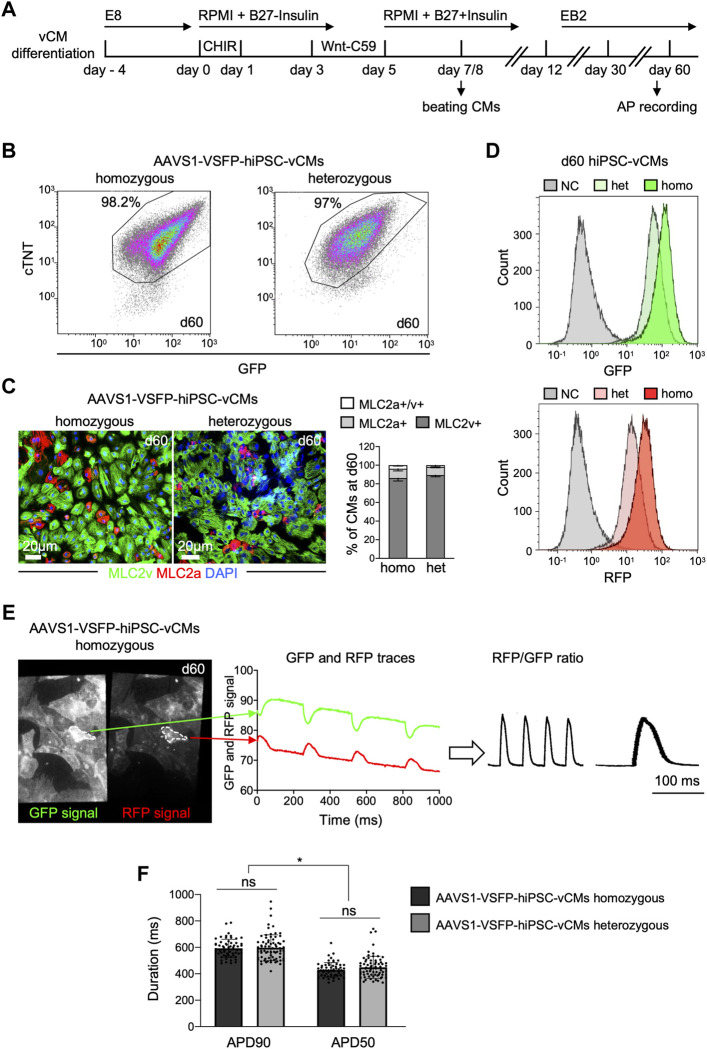
Differentiation and optical AP recordings of AAVS1-VSFP-hiPSC-derived ventricular CMs (AAVS1-VSFP-hiPSC-vCMs). **(A)** Schematic representation of the protocol used for differentiation of hiPSCs into ventricular CMs (vCMs). CHIR: CHIR-98014. **(B)** Representative flow cytometry plots showing d60 (day 60) homozygous and heterozygous AAVS1-VSFP-hiPSC-vCMs immunostained with antibodies against cTNT and GFP. **(C)** Left panel, representative images of d60 homozygous and heterozygous AAVS1-VSFP-hiPSC-vCMs immunostained with antibodies against MLC2v (green) and MLC2a (red). Nuclei were labeled with DAPI (blue). Right panel, quantification of MLC2a+, MLC2a+/v+, and MLC2v+ AAVS1-VSFP-hiPSC-vCMs at day 60. Data are mean ± SD; N = 618 homozygous and N = 444 heterozygous AAVS1-VSFP-hiPSC-vCMs from *n* = 3 differentiations per line. **(D)** Live cytometry analysis of GFP and RFP expression in d60 hetero- (het) and homozygous (homo) AAVS1-VSFP-hiPSC-vCMs. d60 vCMs derived from a control hiPSC line were used as a negative control (NC, gray). **(E)** Spontaneous optical AP measurement of ventricular CMs. Representative GFP and RFP images of d60 homozygous AAVS1-VSFP-hiPSC-vCMs. White dotted lines represent region of interest (ROI) used to quantify the GPF and RFP fluorescence signal (left panel). Background-corrected GFP and RFP fluorescence signals derived from the ROI are depicted (middle panel). The APs are calculated as RFP/GFP ratio (right panel). **(F)** Quantification of APD90 and APD50 in both homo- and heterozygous AAVS1-VSFP-hiPSC-vCMs at day 60 at spontaneous beating. Data are mean ± SD; *N* = 61 homozygous and *N* = 74 heterozygous AAVS1-VSFP-hiPSC-vCMs from *n* = 3 differentiations per line; *p < 0.0001 (Kruskal-Wallis test).

To determine whether VSFP was stably expressed in hiPSC-derived CMs, both homozygous and heterozygous AAVS1-VSFP-hiPSCs were differentiated into monolayers of ventricular CMs using a modified Wnt/β-catenin signaling-based protocol (Foo et al., 2018) (Figure 1A). At day 15, the percentage of cells positive for the CM marker cardiac troponin T (cTNT) was ∼90% in both lines, as detected by flow cytometry (Supplementary Figure S2A), suggesting efficient CM differentiation. The CM purity of homozygous and heterozygous lines was over 97% at day 60 (Figure 1B), when electrophysiological analysis was performed. Immunostaining for the ventricular isoform of the myosin light chain-2 (MLC2v) and the atrial isoform of the myosin light chain-2 (MLC2a) showed that at day 60 around 90% of the CMs were positive for MLC2v, confirming ventricular identity (Figure 1C). The VSFP fluorescence signal intensity increased gradually during cardiac differentiation and was higher in homozygous CMs when compared to heterozygous CMs (Supplementary Figure S2B and Figure 1D). Furthermore, the VSFP signal could be detected at the cell membrane, exhibiting an enrichment at the cell-cell contacts (Supplementary Figure S2C and Supplementary Video S1).

For optical AP recordings, 60 days-old AAVS1-VSFP-hiPSC-vCMs were dissociated and reseeded on fibronectin-coated dishes. Emission from red and green fluorescent proteins was simultaneously recorded at an inverted fluorescence microscope equipped with an image splitter and a high-speed, high-sensitivity sCMOS camera; the RFP/GFP ratio was then used to derive AP traces in individual AAVS1-VSFP-hiPSC-vCMs (Figure 1E and Supplementary Figure S2D). Of note, although the fluorescence signals decreased slightly over the course of the recording time due to photobleaching, the RFP/GFP ratio remained stable due to the ratiometric imaging principle, allowing repeated measurements in a single cell. We determined the AP duration at 90% (APD90) and 50% repolarization (APD50) of spontaneously beating vCMs derived from homozygous and heterozygous AAVS1-VSFP-hiPSCs and obtained similar results from both lines (Figure 1F), indicating that VSFP insertion in one *AAVS1*-allele induced a strong expression of the voltage indicator sufficient to measure APs and both lines could be equally used to assess electrophysiological properties of CMs.

**FIGURE 2:**
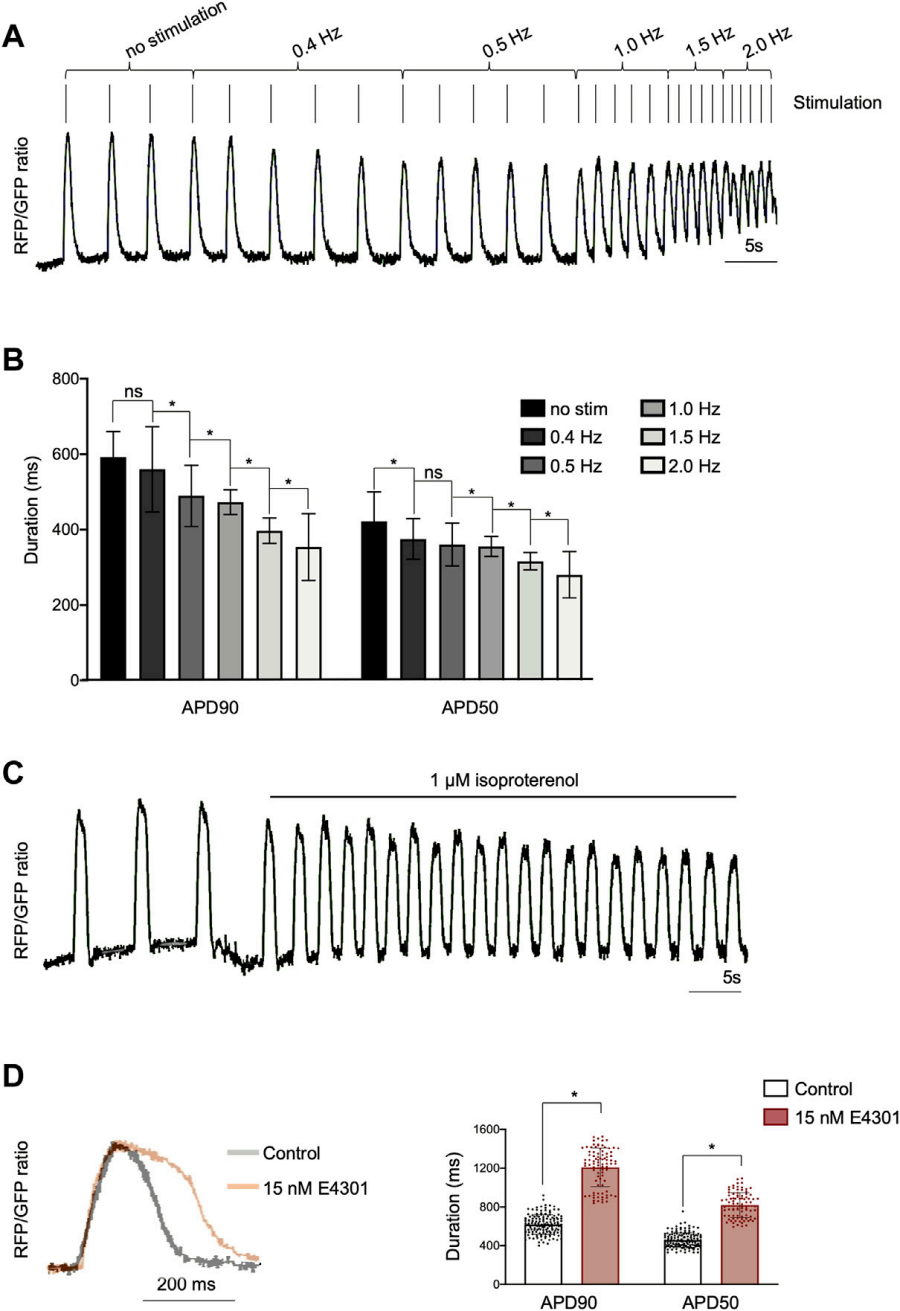
Optical imaging of APs in AAVS1-VSFP-hiPSC-vCMs under electrical stimulation and drug treatment. **(A)** AP traces of homozygous d60 AAVS1-VSFP-hiPSC-vCMs with increasing electrical stimulation from 0.4 Hz to 2 Hz. **(B)** Quantification of APD90 and APD50 in d60 homozygous AAVS1-VSFP-hiPSC-vCMs under electrical stimulation from 0.4 Hz to 2 Hz. No stim: no stimulation. Data are mean ± SD; *N* = 184 vCMs from *n* = 3 differentiations; *p < 0.005 (Kruskal-Wallis test). **(C)** AP traces of homozygous d60 AAVS1-VSFP-hiPSC-vCMs before and after 1 μM isoproterenol treatment. **(D)** AP traces (left panel) and quantification of APD90 and APD50 (right panel) in d60 homozygous AAVS1-VSFP-hiPSC-vCMs with or without (control) 15 nM E4031 treatment. Data are mean ± SD; *N* = 88 with E4031 and *N* = 144 without treatment (control) homozygous vCMs, *n* = 3 differentiations; *p < 0.0001 (Mann-Whitney test).

To demonstrate that AAVS1-VSFP was capable of capturing changes in AP duration, electrical stimulation was applied (50 V, 5 ms) at increasing rates from 0.4 to 2 Hz. Optical AP recordings showed that AAVS1-VSFP-hiPSC-vCMs faithfully responded to pacing and that the AP duration was negatively correlated with the stimulation rate, with a significant reduction of APD90 and APD50 at higher pacing frequencies (Figures 2A,B). We additionally tested the utility of our optical AP recording approach in pharmacological assays. Spontaneously beating AAVS1-VSFP-hiPSC-vCMs treated with isoproterenol, a non-selective β-adrenoreceptor agonist, showed an expected increase in AP frequency (Figure 2C). On the other hand, treatment with the hERG potassium channel blocker E4031 resulted in longer AP duration (Figure 2D).

### Optical AP recordings in AAVS1-VSFP-hiPSC-derived atrial (AAVS1-VSFP-hiPSC-aCMs) and nodal cardiomyocytes (AAVS1-VSFP-hiPSC-nCMs)

**FIGURE 3:**
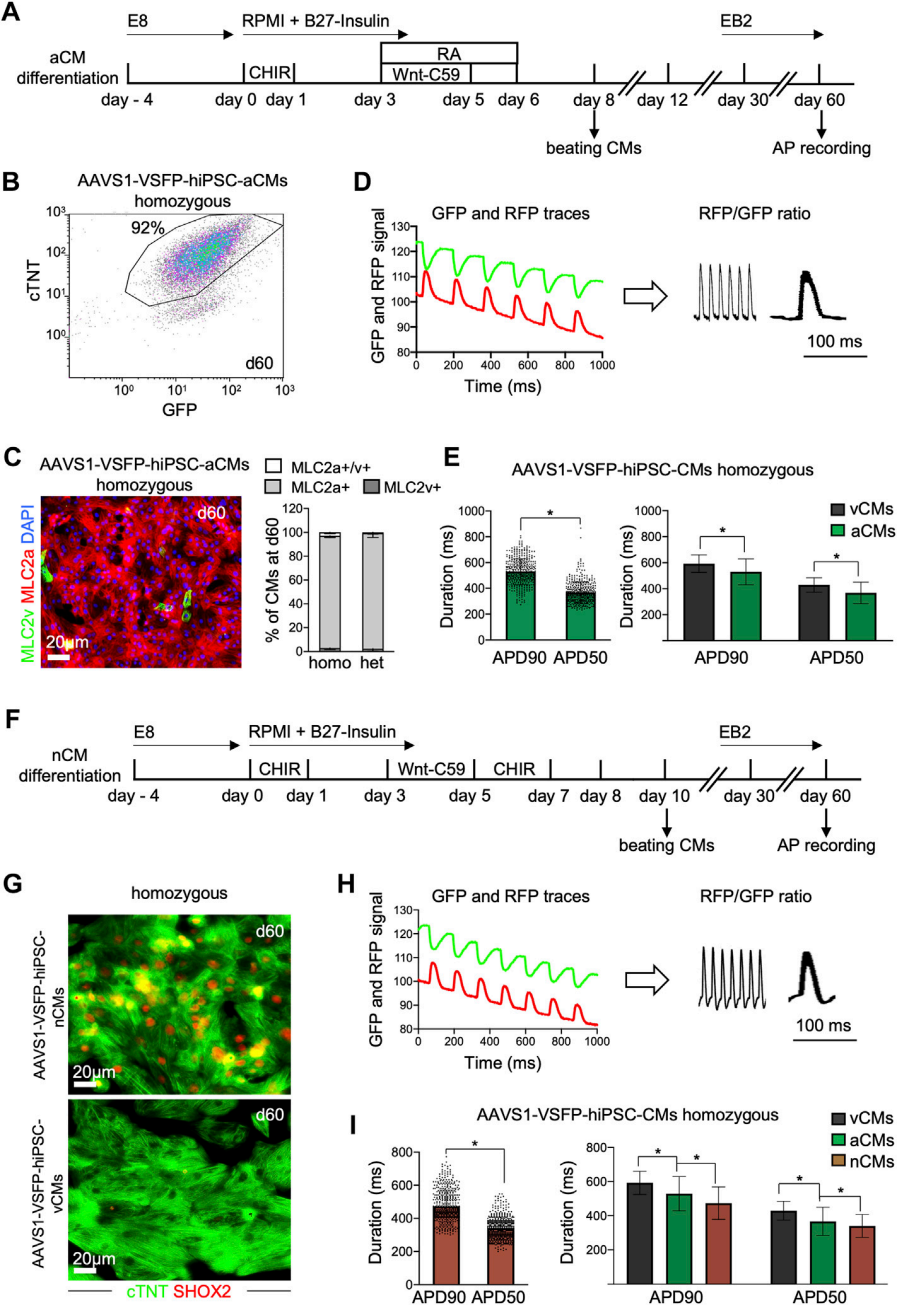
Differentiation and optical AP recordings of AAVS1-VSFP-hiPSC-derived atrial and nodal CMs (AAVS1-VSFP-hiPSC-aCMs/nCMs). **(A)** Schematic representation of the protocol used for differentiation of hiPSCs into atrial CMs (aCMs). CHIR: CHIR-98014; RA: retinoic acid. **(B)** A representative flow cytometry plot showing d60 homozygous AAVS1-VSFP-hiPSC-aCMs immunostained with antibodies against cTNT and GFP. **(C)** Left panel, a representative image of d60 homozygous AAVS1-VSFP-hiPSC-aCMs immunostained with antibodies against MLC2v (green) and MLC2a (red). Nuclei were labeled with DAPI (blue). Right panel, quantification of MLC2a+. MLC2a+/v+ and MLC2v+ AAVS1-VSFP-hiPSC-aCMs at day 60. Data are mean ± SD; *N* = 507 homozygous and N = 378 heterozygous AAVS1-VSFP-hiPSC-aCMs from *n* = 3 differentiations per line. **(D)** Spontaneous optical AP measurement of atrial CMs. Background-corrected GFP and RFP fluorescence traces of d60 homozygous AAVS1-VSFP-hiPSC-aCMs (left panel). The APs are calculated as RFP/GFP ratio (right panel). **(E)** Quantification of APD90 and APD50 in d60 homozygous AAVS1-VSFP-hiPSC-aCMs at spontaneous beating (left panel) and comparison of APD90 and APD50 between AAVS1-VSFP-hiPSC-vCMs and -aCMs (right panel). Data are mean ± SD; *N* = 61 vCMs from *n* = 3 differentiations and *N* = 434 aCMs from *n* = 5 differentiations; *p < 0.0001 (Mann-Whitney test). **(F)** Schematic representation of the protocol used for differentiation of hiPSCs into nodal CMs (nCMs). CHIR (d0-d1): CHIR-98014, CHIR (d5-d7): CHIR-99021. **(G)** Representative images of d60 homozygous AAVS1-VSFP-hiPSC-nCMs and -vCMs immunostained with antibodies against SHOX2 (red) and cTNT (green). **(H)** Spontaneous optical AP measurement of nodal CMs. Background-corrected GFP and RFP fluorescence traces of d60 homozygous AAVS1-VSFP-hiPSC-nCMs at spontaneous beating (left panel). The APs are calculated as RFP/GFP ratio (right panel). **(I)** Quantification of APD90 and APD50 in d60 homozygous AAVS1-VSFP-hiPSC-nCMs (left panel) and comparison of APD90 and APD50 between AAVS1-VSFP-hiPSC-vCMs, -aCMs and -nCMs (right panel). Data are mean ± SD; *N* = 61 vCMs from *n* = 3 differentiations, *N* = 434 aCMs from *n* = 5 differentiations, *N* = 476 nCMs from *n* = 5 differentiations; *p < 0.0001 (Mann-Whitney test, left panel; Kruskal-Wallis test, right panel).

Recent advances in cardiac differentiation have facilitated the *in vitro* generation of subtype-specific CMs, a great asset for both *in vitro* modeling and potential cell therapy approaches (Zhao et al., 2020). To verify that our genetically encoded voltage indicator could detect subtype-specific differences in APs, we differentiated AAVS1-VSFP-hiPSCs towards atrial and nodal CMs and analyzed APs in these cells (Figure 3). We utilized a Wnt-based protocol in combination with retinoic acid (RA) to generate atrial CMs (Figure 3A) (Cyganek et al., 2018; Foo et al*.*, 2018). Similar to the ventricular protocol, the atrial differentiation resulted in high CM differentiation efficiency of ∼90%, as detected by flow cytometry at day 15 and 60 (Supplementary Figure S3A and Figure 3B). More than 95% of the CMs were positive for MLC2a in both homozygous and heterozygous lines (Figure 3C, Supplementary Figure S3B), suggesting a highly efficient CM subtype specification. Next, we evaluated the VSFP expression in AAVS1-VSFP-hiPSC-aCMs by live cell imaging and flow cytometry and obtained comparable results as in AAVS1-VSFP-hiPSC-vCMs (Supplementary Figures S3C, S3D). Functionally, optical AP measurements (Figure 3D) showed significantly shorter APD90 and APD50 in 60-days old AAVS1-VSFP-hiPSC-aCMs when compared to AAVS1-VSFP-hiPSC-vCMs (Figure 3E), confirming a typical atrial-like electrophysiological phenotype (Cyganek et al., 2018). Similar APD90 and APD50 results were obtained in the heterozygous line (Supplementary Figure S3E).

For the generation of the nodal CMs, we combined a Wnt-based protocol promoting ventricular CM subtype specification (Foo et al*.*, 2018) with an additional Wnt-activation step from day 5–7, as previously described (Ren et al., 2019) (Figure 3F). At day 15, the percentage of cTNT + cells was around 90%, as verified by flow cytometry (Supplementary Figure S3F), indicating efficient CM differentiation. The CM purity was over 98% at day 60 (Supplementary Figure S3F), when APs were recorded. Immunostaining of AAVS1-VSFP-hiPSC-nCMs revealed that over 95% of the cTNT + CMs were also positive for the nodal lineage marker SHOX2 (Figure 3G and Supplementary Figure S3G), suggesting efficient specification into nodal CM subtype. The VSFP expression in AAVS1-VSFP-hiPSC-nCMs was similar to ventricular and atrial cells, as supported by live cell imaging and flow cytometry (Supplementary Figures S3H, S3I). Further functional analysis demonstrated (Figure 3H) that the APD90 and APD50 were significantly shorter in nodal when compared to atrial and ventricular CMs (Figure 3I), which was consistent with a typical nodal-like electrophysiological phenotype described previously (Ma et al., 2011; Burridge et al., 2014; Jouni et al., 2015; Yechikov et al., 2016; Poulin et al., 2019; Zhang et al., 2019; Liang et al., 2020; Poulin et al., 2021).

Taken together, using hiPSCs carrying a stably integrated VSFP in the *AAVS1* locus, we could successfully record optical APs in ventricular, atrial, and nodal CMs generated with subtype-specific differentiation protocols.

### AAVS1-VSFP enables optical monitoring of APs in single hiPSC-derived cardiomyocytes within 3D heart patches

**FIGURE 4:**
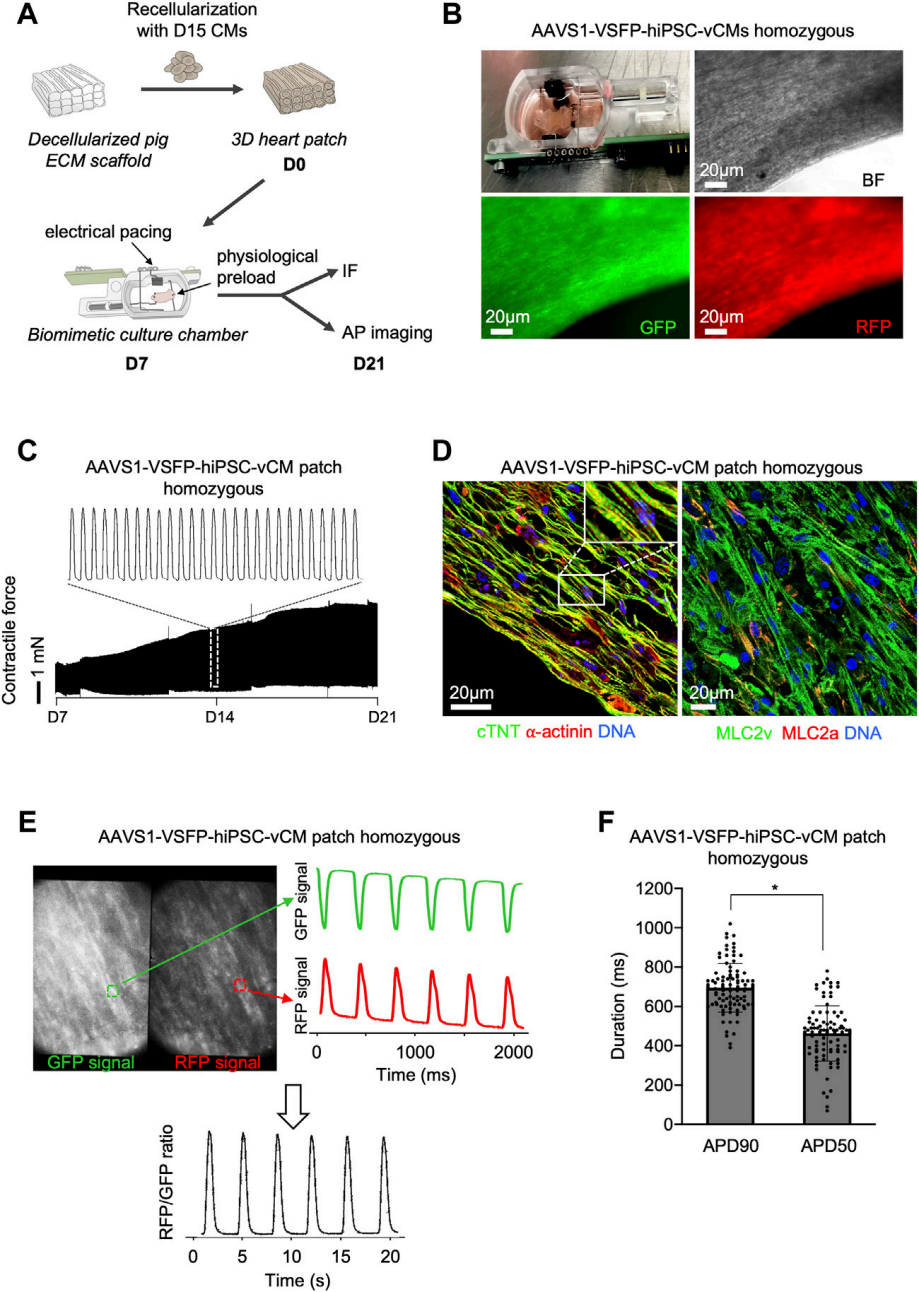
Optical AP recordings in single ventricular AAVS1-VSFP-hiPSC-CMs within 3D heart patches. **(A)** Graphic representation of the experimental setup for 3D culture of hiPSC-derived CMs. Day 15 hiPSC-derived ventricular CMs were reseeded on decellularized pig ECM scaffold, which were transferred to biomimetic culture chambers und cultured under mechanical load and electrical pacing for 2 weeks allowing continuous recording of the contractile forces. IF: immunofluorescence. **(B)** An image depicting the 3D heart patch in the biomimetic culture chamber (upper panel left). Brightfield (BF), GFP and RFP live images of the 3D scaffold recellularized with AAVS1-VSFP-hiPSC-vCMs. **(C)** Representative contractile force traces from 3D heart patches reseeded with AAVS1-VSFP-vCMs during 2 weeks of culture in biomimetic chamber (lower panel). White dotted rectangle presents a zoom into the contractile force performance at day 14 (upper panel). **(D)** Representative images of a 3D heart patch after 2 weeks in culture immunostained with antibodies against cTNT (green) and ⍺-actinin (red) (left) as well as MLC2v (green) and MLC2a (red) (right). Nuclei were labeled with DAPI (blue). White rectangle represents a zoom into the specific region of cTNT and ⍺-actinin staining. **(E)** Spontaneous optical AP recording in a 3D heart patch. Representative GFP and RFP images of a 3D construct. Selected fields (white dotted rectangles) represent a ROI used to quantify the GFP and RFP fluorescence signal (left panel). Background-corrected GFP and RFP fluorescence signals derived from the ROI (right panel). The APs were calculated as RFP/GFP ratio (lower panel). **(F)** Quantification of APD90 and APD50 in 3D heart patches. Data are mean ± SD; N = 96 CMs from *n* = 3 patches; *p < 0.0001 (Mann-Whitney test).

We next explored the possibility of assessing APs in single CMs in a 3D context that provides a physiological environment similar to an *in vivo* setting. We used a recently established 3D model by repopulating decellularized scaffolds from porcine left ventricle heart slices with early 15-days old hiPSC-vCMs (Krane et al., 2021) (Figure 4A). After integration of the CMs into the extracellular matrix (ECM), the heart patches were cultured in biomimetic chambers under mechanical loading and electrical stimulation for 2 weeks (Figure 4B and Supplementary Video S2). An increase of contractile force was observed (Figure 4C), suggesting electromechanical maturation of the CMs. Notably, when compared to 2D, hiPSC-CMs in 3D displayed a more elongated CM shape, a feature of adult CMs, indicating further maturation (Figure 4D). Moreover, the 3D CMs exhibited highly-organized sarcomeres as visualized by α-actinin and cTNT immunostaining and were mainly of ventricular identity (Figure 4D). The GFP and RFP signals in single CMs within the 3D heart patches could be clearly recorded, allowing the calculation of the RFP/GFP ratio as membrane potential (Figure 4E), which could be used in downstream analysis to determine APD90 and APD50 values (Figure 4F), demonstrating that the VSFP knock-in system enables a high-fidelity measurement of APs in complex 3D environments, and highlighting its potential for use in future *in vivo* studies.

## Discussion

Arrhythmias are a common cause of sudden cardiac death, which can be primarily attributed to rare monogenic defects affecting electric excitation and contraction, or more frequently, secondarily, to common multifactorial diseases (e.g., heart failure, myocardial infarction) (Srinivasan and Schilling, 2018; Lucena, 2019; Scrocco et al., 2021). With the advent of hiPSC technology, it became possible to model human cardiac development and diseases in a dish. Furthermore, with a focus on electrophysiological characterization, hiPSC-derived CMs offer a system to investigate the susceptibility to cardiac arrhythmias in drug screening and cardiac safety assays (Gintant et al*.*, 2017; Ovics et al., 2020).

In this study, we have functionally validated our recently generated AAVS1-VSFP-hiPSC lines carrying a GEVI in the *AAVS1* safe harbor locus, revealing sufficient VSFP expression upon CM differentiation. These lines provide a powerful tool for high-throughput recording of APs and can be used in cardiac disease models, drug screening and toxicity testing. Our results demonstrate that AAVS1-VSFP is capable of robustly reporting AP dynamics under a wide range of conditions, including electrical pacing and drug treatment. In addition to recordings in 2D monolayers, we were able to simultaneously acquire membrane potentials of single CMs in a 3D environment, allowing for population studies that could facilitate the development and evaluation of more functionally mature, biologically relevant engineered cardiac tissues.

In comparison with previously reported techniques for electrophysiological phenotyping of pluripotent stem cell-derived CMs, the AAVS1-VSFP-hiPSCs reporter cell lines offer several advantages. First, in contrast to traditional single-cell patch-clamp methodology, the optical approach increases the throughput multiple times and does not rely on complex equipment setup. Furthermore, it allows sequential investigation of the same cells during prolonged time periods to follow AP dynamics. The next advantage of the AAVS1-VSFP reporter is that, unlike voltage-sensitive synthetic dyes such as FluoVolt (Broyles et al., 2018), which require loading each time before imaging, it consistently detects membrane potential with virtually no processing time and no phototoxicity. Finally, optical AP acquisition using VSFP voltage sensor is based on the ratiometric readout and, thus, less susceptible to cell movement artefacts and photobleaching. In contrast, the genetically encoded voltage indicator ArcLight, which has been recently integrated into the *AAVS1* locus and successfully used to image APs in hiPSC-CMs (Shinnawi et al., 2015; Song et al., 2015; Sun et al., 2020), relies only on relative membrane potential changes and is therefore more prone to movement artefacts due to beating of CMs.

The VSFP expression was stable in AAVS1-VSFP-hiPSCs and increased during cardiac differentiation, enabling long-term monitoring of the AP dynamics. This was consistent with previously described knock-in of the ArcLight voltage sensor into the *AAVS1* locus, exhibiting a higher reporter expression in CMs when compared to undifferentiated hiPSCs (Sun et al., 2020). The observed APs were similar to those reported for hiPSC-CMs transduced with lentiviral vectors encoding VSFP (Kaestner et al., 2015; Chen et al., 2017; Goedel et al., 2018). Importantly, insertion of the reporter in one *AAVS1* allele resulted in expression levels sufficient to detect and measure APs in hiPSC-derived CMs. Although the VSFP signal intensity was lower in the heterozygous than in homozygous AAVS1-VSFP-hiPSCs, the AP results from both lines were comparable, indicating that these two lines could be equivalently used in assessing AP characteristics.

Taking advantage of CRISPR/Cas9 technology, single or multiple mutations related e.g. to hereditary arrhythmia could be introduced into the AAVS1-VSFP-hiPSC lines to establish novel disease models, which could be deployed in mechanistic studies or pharmacological assays. Additionally, these cell lines might also be beneficial for functional investigation of other hiPSC-derived cell types, e.g. in excitation studies in neurons.

Although optical recordings do not fully replace classical patch-clamp recordings and it is not possible to determine absolute parameters of transmembrane potentials, still, the AAVS1-VSFP system is able to detect subtle alterations in AP duration associated with increasing electrical stimulation or drug application. Furthermore, it can be used to determine AP duration differences in distinct CM subtypes.

In conclusion, in this study, we generated a tool for high-throughput optical AP recordings based on the knock-in of the genetically encoded VSFP into the *AAVS1* safe harbor locus allowing for assessment of electrophysiological properties of hiPSC-CMs in both 2D and 3D environments.

## Materials and methods

### Human induced pluripotent stem cell culture

hiPSCs (Control hiPSCs, ID MRIi003-A; heterozygous AAVS1-VSFP-hiPSCs, ID MRIi003-A-5; and homozygous AAVS1-VSFP-hiPSCs, ID MRIi003-A-6) were cultured on Geltrex-coated (Thermofisher Scientific, A14133-02) tissue culture plates (Falcon, 353001 and 353004) in Essential 8 medium (Thermofisher Scientific, A1517001) containing 0.5% Penicillin/Streptomycin (Thermofisher Scientific, 15140122) under standard culture conditions (37°C, 5% CO_2_). Medium was changed daily. Cells were passaged at a 1:14 ratio every 4–5 days using 0.5 mM EDTA (Thermofisher Scientific, AM9260G). After passaging, the medium was supplemented with 10 μM Thiazovivin (Sigma-Aldrich, SML1045) for 24 h to promote survival.

### Cloning of the donor pAAVS1-p-CAG-VSFP-polyA construct

The cloning strategy of the donor plasmid pAAVS1-p-CAG-VSFP-polyA has been described in Zhang et al., 2022 Primers used for cloning and sequencing of the pAAVS1-p-CAG-VSFP-polyA construct are listed in Supplementary Table S1.

### CRISPR/Cas9-mediated insertion of the CAG-VSFP cassette into the *AAVS1* locus in hiPSCs using 4D nucleofection

CRISPR/Cas9-mediated insertion of the CAG-VSFP cassette into the *AAVS1* locus (intron 1 of the *PPP1R12C* gene) in control hiPSCs was achieved using the 4D Nucleofection system. Briefly, control hiPSCs were dissociated with Accutase (Thermofisher Scientific, A1110501) and 1x10^6^ cells were nucleofected with 1 µg pXAT2 plasmid (containing Cas9-nuclease and *AAVS1* locus specific sgRNA GGG​GCC​ACT​AGG​GAC​AGG​AT; Addgene, 80494) and 3 µg donor construct (pAAVS1-p-CAG-VSFP-polyA) following the Lonza 4D Nucleofector basic protocol for human stem cells. After transfection, hiPSCs were reseeded on Matrigel-coated (BD, 354277) 6 well-plates (Nunclon, 150687) in mTeSR1 (Stemcell Technologies, 05854) containing 10 μM Thiazovivin to improve cell survival. Next day, the medium was exchanged by mTeSR1 containing no Thiazovivin. 48 h after transfection, 0.2 μg/ml puromycin (Calbiochem, 540411) was added to the cells for 7 days for selection of targeted clones. When the hiPSC colonies were big enough, cells were dissociated with Accutase and replated at low density (1,000 cells per dish) on Matrigel-coated 10 cm dishes (Nunc, 150350). After 7–10 days, colonies derived from single clones were cut into two halves, one half was used for further expansion in a 96-well plate (Nunclon, 161093) and the other half was subjected to PCR screening. Insertion was verified by Sanger sequencing (Eurofins MWG Operon). Primers used for PCR amplification of the *AAVS1* locus before and after CAG-VSFP insertion are listed in Supplementary Table S1. Primers used for sequencing of the AAVS1-CAG-VSFP-hiPSC clones are described in Zhang et al., 2022. The generated AAVS1-CAG-VSFP hiPSC lines were confirmed to have a normal karyotype (Institute of Human Genetics of the Klinikum rechts der Isar, Technical University of Munich).

### Differentiation of hiPSCs into ventricular cardiomyocytes

Differentiation into vCMs was induced by modulation of Wnt/β-catenin signalling, following a protocol described by Foo et al., 2018. Briefly, hiPSCs were dissociated with 0.5 mM EDTA and seeded on Geltrex-coated 24-well plates (Nunclon, 142475) at a density of 1x10^5^ cells per well. Upon reaching 95% confluence after 3–4 days, vCM differentiation was induced on day 0 by changing to RPMI1640 (Thermofisher Scientific, 21875034) containing B27 insulin minus (Thermofisher Scientific, A1895601) (defined as basal cardiac differentiation medium) supplemented with 1 µM CHIR-98014 (Selleckchem, S2745). On day 1, medium was replaced with basal cardiac differentiation medium. On day 3, medium was replaced with basal cardiac differentiation medium supplemented with 2 µM Wnt-C59 (Selleckchem, S7037). From day 5 on, medium was replaced with RPMI1640 medium containing B27 insulin plus. Spontaneous beating typically initiated on days 7–8.

### Differentiation of hiPSCs into atrial and nodal cardiomyocytes

For both aCM and nCM differentiation, we modified the Wnt-based vCM protocol (Foo et al*.*, 2018) according to recent reports describing atrial (Cyganek et al*.*, 2018)and nodal (Ren et al*.*, 2019) cardiomyocyte differentiation.

aCM differentiation was induced on day 0 by changing to basal cardiac differentiation medium supplemented with 1 µM CHIR-98014. On day 1, medium was replaced with basal cardiac differentiation medium. On day 3, medium was replaced with basal cardiac differentiation medium plus 2 µM Wnt-C59 and 1 µM retinoic acid (RA; Sigma-Aldrich, R2625). At day 5, medium was replaced with basal cardiac differentiation medium with 1 µM RA. On day 6, medium was exchanged by basal cardiac differentiation medium. From day 8 on, medium was replaced with RPMI1640 medium containing B27 insulin plus. Spontaneous beating typically initiated on days 8–10.

For nCM lineage, the differentiation from day 0 to day 5 was the same as for vCMs. At day 5, the medium was replaced with basal cardiac differentiation medium containing 3 µM CHIR-99021 (Axon, CT99021). On day 7 and 9, the medium was exchanged by the basal cardiac differentiation medium. From day 11 on, medium was replaced with RPMI1640 medium containing B27 insulin plus. Spontaneous beating initiated on days 10–11.

### Long-term maintenance of cardiomyocytes

At day 20–24 of differentiation, beating areas were manually dissected and transferred to fibronectin-coated plates and maintained in EB2 medium consisting of DMEM/F-12 (Thermofisher Scientific, 11320033) supplemented with 2% fetal bovine serum (FBS, Thermofisher Scientific, 16141079), 1% non-essential amino acids (Thermofisher Scientific, 11140050), 0.5% Penicillin-Streptomycin (Thermofisher Scientific, 15140122), 1% L-glutamine (Thermofisher Scientific, 25030081) and 0.1 mM β-mercaptoethanol (Sigma-Aldrich, M6250).

### Dissociation of hiPSC-derived cardiomyocytes

For dissociation, hiPSC-CMs were harvested at 15 (d15), 30 (d30) or 60 days (d60) of cardiac differentiation and treated with papain (Worthington Biochemical Corporation, LS003124) following a protocol described by Fischer and colleagues with some modifications (Fischer et al., 2018). Briefly, d15 and d30 hiPSC-CMs were washed twice with 2 mM EDTA in DPBS without Ca^2+^/Mg^2+^ (Sigma-Aldrich, D8537) and then incubated with a solution containing 20 U/mL papain and 1 mM L-cysteine (Sigma-Aldrich, C6852) in DPBS for 30 min at 37°C. The reaction was stopped by addition of one volume of DPBS containing 1 mg/ml trypsin inhibitor (Sigma-Aldrich, T9253) and 40 μg/ml DNAse I (Sigma-Aldrich, DN25). For d60 hiPSC-CMs, the explants were dissected and incubated with papain solution under shaking at 37°C for 30 min. Afterwards, the reaction was stopped by trypsin inhibitor solution. The single cells were collected either for further cultivation in 2D and 3D or immunohistological and flow cytometry analysis.

### Immunohistological analysis

For immunofluorescence staining, cells were fixed with 4% paraformaldehyde (PFA; Sigma-Aldrich, 158127) for 10 min at room temperature (RT) and washed three times with DPBS without Ca^2+^/Mg^2+^. Cells were then permeabilized with 0.25% Triton X-100 (Sigma-Aldrich, X100) for 15 min at RT before washing three times with DPBS, followed by incubation with 10% FBS in DPBS containing 0.1% TritonX-100 (PBST) for 1 h at RT. Primary antibodies against cardiac Troponin (cTNT), α-actinin, MLC2v, MLC2a and SHOX2 were incubated in 1% FBS in PBST overnight at 4°C. After washing five times for 5 minutes with PBST, appropriate secondary antibodies were added in PBST for 1 h at RT. After washing five times for 5 minutes with PBST, Hoechst 33258 was incubated at the final concentration of 5 μg/ml in DPBS for 15 min at RT. After washing twice with DPBS, cells were mounted with coverslips using fluorescence mounting medium (Dako, S3023) and stored at 4°C until imaging with an inverted confocal microscope (Leica DMi8, Leica Microsystems, Wetzlar, Germany). Primary and secondary antibodies are listed in Supplementary Table S2.

### Flow cytometry

For flow cytometry-based acquisition of GFP and RFP fluorescence in live cells, hiPSCs and CMs were dissociated with accutase and papain, respectively. For cTNT and GFP flow cytometry analysis in fixed cells, hiPSC-CMs at day 15, 30 or 60 of differentiation were dissociated with papain and 1x10^6^ cells were fixed in 4% PFA for 15 min at RT. Samples were permeabilized with 0.25% Triton X-100 (Sigma-Aldrich, X100) in DBPS for 15 min and blocked with 10% FBS in PBST for 1 h at RT. Incubation with primary antibodies for cTNT and GFP was carried out in 1% FBS in PBST overnight at 4°C. For detection, fluorescent-dye conjugated secondary antibodies specific to the appropriate species were used for 1 h at RT. Samples were measured on the Gallios flow cytometer (Beckman Coulter, Germany) and data were analyzed using Kaluza software version 1.2 (Beckman Coulter). Primary and secondary antibodies are listed in Supplementary Table S2.

### Porcine extracellular matrix generation and 3D hiPSC-derived cardiomyocytes

For porcine ECM generation, porcine myocardial tissue was obtained from left mid-ventricular transmural sections and immediately placed in PBS (Thermofisher Scientific, 10010015). The tissue was then embedded in 4% agarose in PBS and sectioned to 300 µm thick slices on the vibratome (VT1200S, Leica Biosystems, Germany). Decellularization of the heart slices was performed in lysis buffer (10 mM Tris, 0.1% EDTA, pH 7.4) overnight under agitation on orbital shaker at RT followed by incubation in 0.5% SDS solution for at least 6 h under agitation on orbital shaker at RT. Samples were then washed three times with PBS and incubated in 50% FBS in PBS overnight at 4°C. Then, the ECM slices were washed with PBS and stored in PBS containing 1% Penicillin-Streptomycin up to 3 weeks at 4°C.

For 3D culture of hiPSC-CMs, heart ECM slices were cut to suitable size and small plastic triangles were attached to them with tissue adhesive (Histoacryl, B. Braun 69390) according to the fiber direction. hiPSC-CMs were dissociated at day 15 into single cells using papain and reseeded onto ECM slices placed in cell culture plate inserts (Millipore, PICM03050) in 6 well plates. 1 ml EB2 medium was added underneath the inserts and medium was exchanged every day. Seven days after reseeding, ECM slices with hiPSC-CMs were anchored in biomimetic culture chambers (InVitroSys) and subjected to physiological preload of 1 mN and stimulation at 0.5 Hz (50 mA pulse current, 1 ms pulse duration). The slices were maintained in EB2 medium, which was replaced every other day, on a rocker plate (60 rpm, 15 °C tilt angle) placed in an incubator set at 37°C, 5% CO_2_, 20% O_2_ and 80% humidity. A continuous readout of contraction force was obtained *via* the biomimetic chamber (Fischer et al., 2019; Ou et al., 2019; Krane et al., 2021; Lu et al., 2021). Contractility data were analyzed by LabChart Reader software.

### Action potential imaging

For AP recording in 2D, hiPSC-CMs at day 60 were dissociated into single cells using papain and plated in EB2 medium on a 3.5 cm glass-bottom cell culture micro-dish (MatTeck Corporation, P35G-1.5-14-C) coated with 2 μg/cm^2^ fibronectin (Sigma-Aldrich, F1141). After dissociation, single hiPSC-CMs were cultured in EB2 medium for at least 3 days to ensure a sufficient recovery. Seeding cell density of 80% was optimal to AP recordings. Before imaging, the EB2 medium was exchanged by Tyrode’s solution supplemented with Ca^2+^ (135 mM NaCl, 5.4 mM KCl, 1 mM MgCl_2_, 10 mM glucose, 1.8 mM CaCl_2_, and 10 mM HEPES; pH 7.35). Similarly, for AP recording of hiPSC-CMs in 3D, the heart patches were transferred to Tyrode’s solution in 3.5 cm glass-bottom cell culture micro-dish. To block the rapid component of the delayed rectifying potassium current (I_Kr_) conducted by hERG channel, 15 nM E4031 (Abcam, ab120158) was added to the Tyrode’s solution and the samples were incubated 20 min at 37°C and then APs were recorded at RT. To increase the beating frequency, 1 µM isoproterenol (Sigma, I6504-100 MG), a nonselective β-adrenoreceptor agonist, was applied to spontaneously-beating iPSC-CMs in Tyrode’s solution supplemented with Ca^2+^.

APs were imaged according to the protocol described by Goedel and colleagues, with some modifications (Goedel et al., 2018). Briefly, the glass bottom micro-dishes with the hiPSC-CMs were placed on the stage of an inverted fluorescence microscope (DMI6000B, Leica Microsystems, Wetzlar, Germany), equipped with an image splitter, the appropriate filter sets (a 480/40 nm band-pass excitation filter combined with a 500 nm long-pass dichroic mirror in the microscope, and a 568 nm long-pass dichroic mirror combined with 520/28 nm and 630/75 nm band-pass emission filters in the image splitter), a HCX PL APO 63X/1.4–0.6 oil immersion objective (Leica Microsystems) as well as a Zyla V sCMOS camera (Andor Technology, Belfast, United Kingdom) capable of high-speed imaging at a high sensitivity. Images were acquired using the Andor Solis Acquisition and Analysis software. Field stimulation electrodes (RC-37FS, Warner Instruments, Hamden, CT, United States) were connected to a stimulus generator (HSE Stimulator P, Hugo Sachs Elektronik, March-Hugstetten, Germany) providing depolarizing pulses (50 V, 5 ms duration). Electrical stimulation was performed at increasing frequencies ranging from 0.4 to 2 Hz.

Imaging settings (illumination intensity, camera gain, binning) were adjusted to achieve an optimal signal-to-noise ratio, while avoiding pixel saturation. Imaging rates were set to 100 Hz. ImageJ (National Institutes of Health, Bethesda, MD) was used to quantify fluorescence over single cells and over background regions in both GFP and RFP channels. Subsequent analysis was performed in RStudio (RStudio Team, 2020) using custom-written scripts. After subtraction of background fluorescence from both GFP and RFP channels, the RFP/GFP ratio representing APs was calculated. After manual selection of the starting points and the peaks of the APs, the transient duration at 90% (APD90) or 50% (APD50) were automatically determined by the script.

### Statistical analysis

Data are presented as mean ± SD unless otherwise specified. Statistical analyses were performed with GraphPad Prism 8. Data from two experimental groups that were not normally distributed were compared by Mann-Whitney test. Normally distributed data from more than two experimental groups were compared using one-way analysis of variance (ANOVA); for nonparametric testing of more than two groups, the Kruskal-Wallis test was applied. *p*-value < 0.05 was considered statistically significant.

## Data Availability

The raw data supporting the conclusion of this article will be made available by the authors, without undue reservation.

## Supplementary material

Supplementary Figure S1 “Generation of the voltage sensor hiPSC lines by insertion of the VSFP into the AAVS1 locus using CRISPR/Cas9“

Supplementary Figure S2 “Characterization and optical AP recordings of AAVS1-VSFP-hiPSC-vCMs”

Supplementary Figure S3 “Characterization and optical AP recordings of AAVS1-VSFP-hiPSC-aCMs and -nCMs”

Supplementary Video 1a “Movie of d60 homozygous AAVS1-VSFP-hiPSC-vCMs (GFP)”

Supplementary Video 1b “Movie of d60 homozygous AAVS1-VSFP-hiPSC-vCMs (RFP)”

Supplementary Video 2a “Movie of AAVS1-VSFP-hiPSC-vCM 3D heart patches at day 21 after reseeding (GFP)”

Supplementary Video 2b “Movie of AAVS1-VSFP-hiPSC-vCM 3D heart patches at day 21 after reseeding (RFP)”
