# Supplementary figures and images for: High-throughput optical action potential recordings in hiPSC-derived cardiomyocytes with a genetically encoded voltage indicator in the AAVS1 locus
Source: Front Cell Dev Biol. 2022 Oct 7;10:1038867. doi: 10.3389/fcell.2022.1038867 (PMC9585323; doi:10.3389/fcell.2022.1038867)

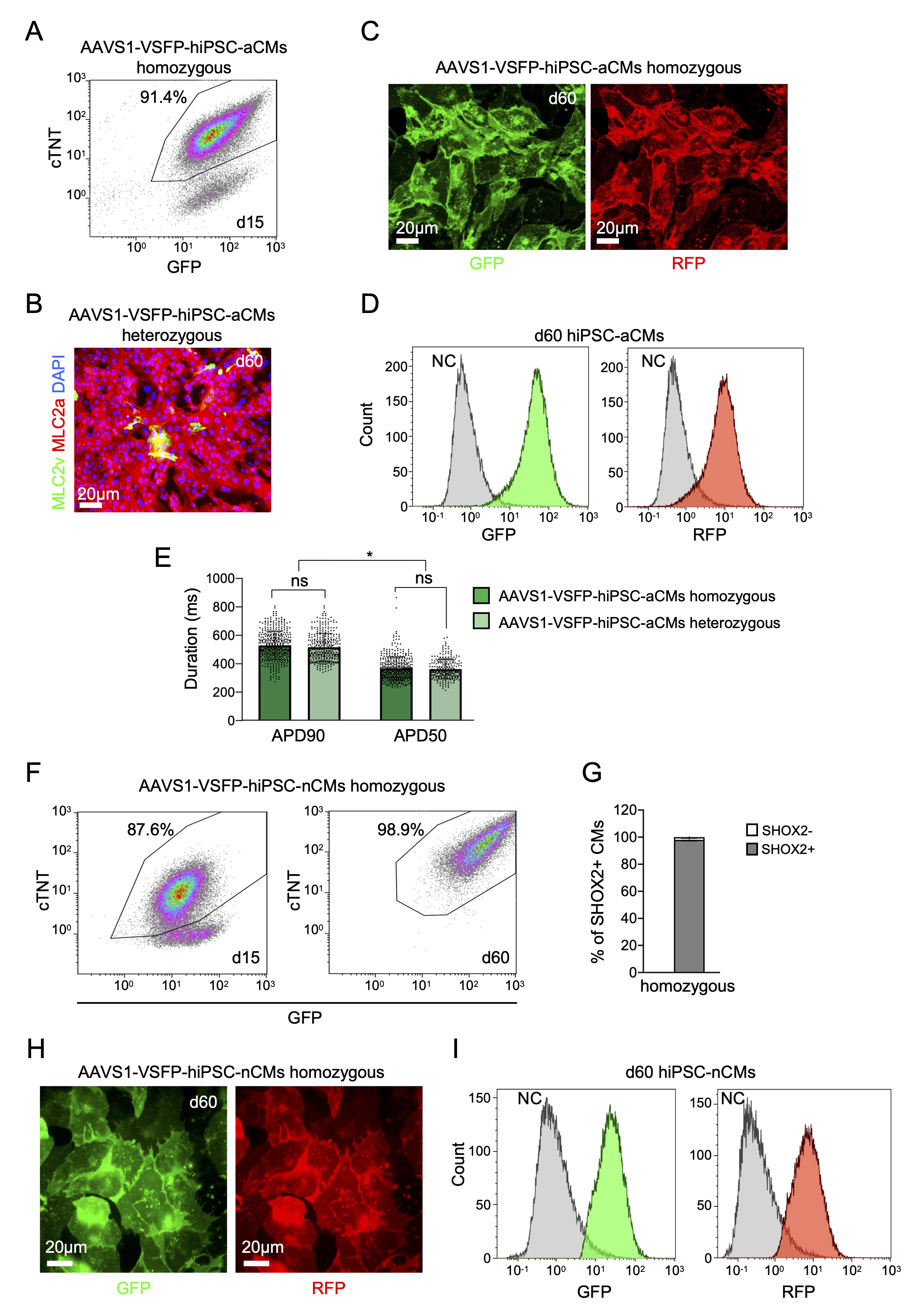

Supplement: Supplementary file 1 [file Image3.TIFF]

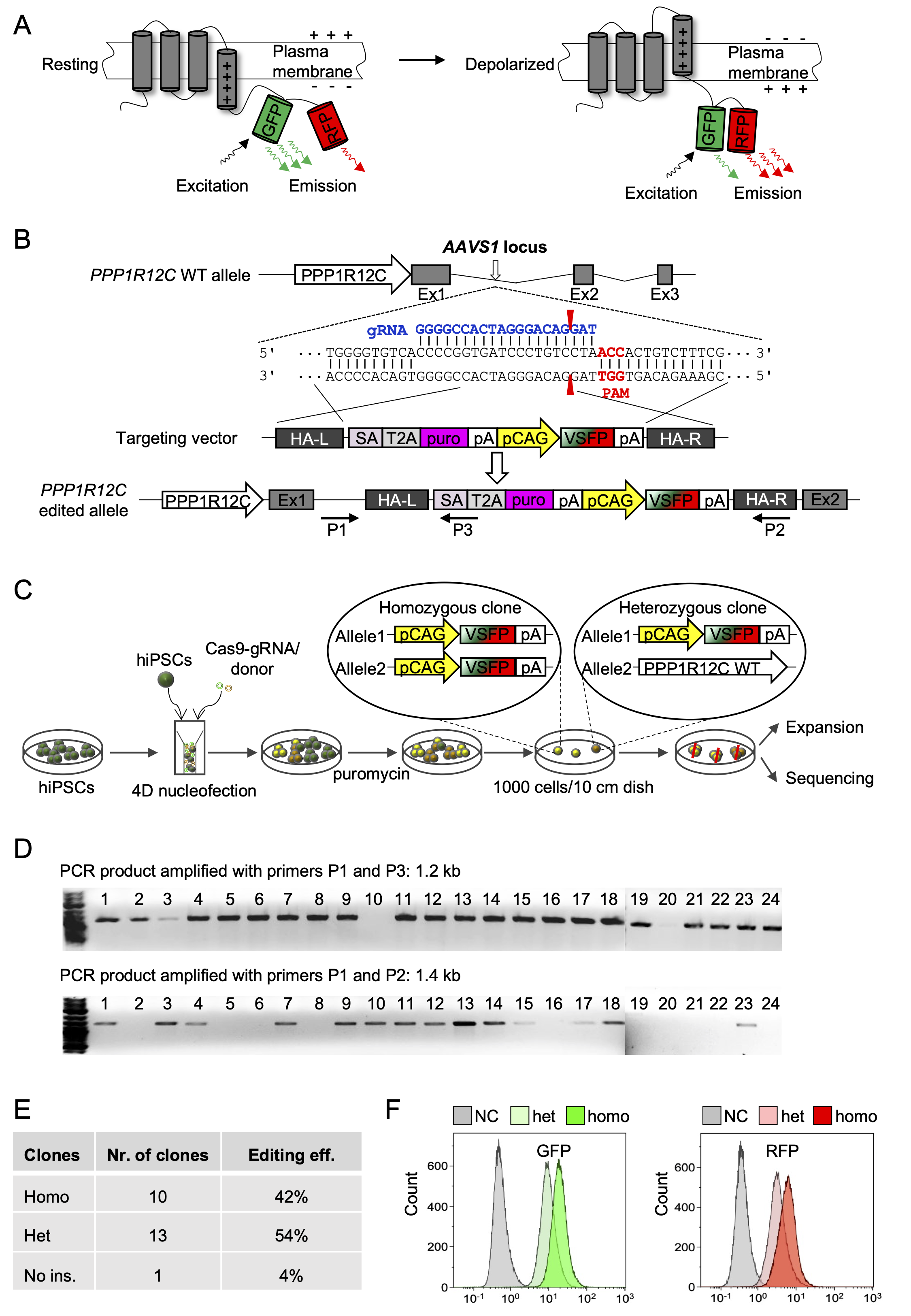

Supplement: Supplementary file 2 [file Image1.TIFF]

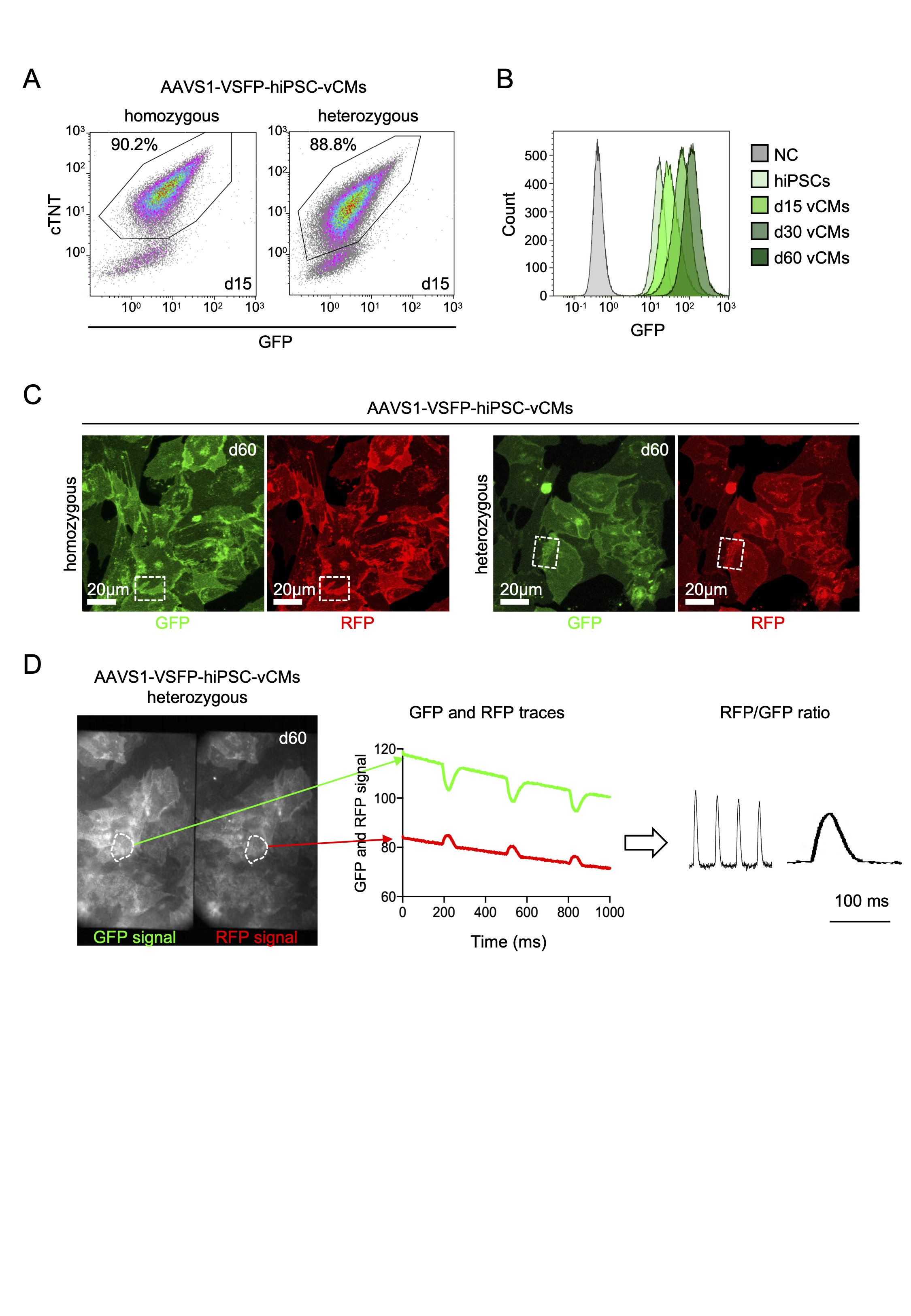

Supplement: Supplementary file 8 [file Image2.TIFF]
