# Supplementary material for: High-throughput optical action potential recordings in hiPSC-derived cardiomyocytes with a genetically encoded voltage indicator in the AAVS1 locus
Source: Front Cell Dev Biol. 2022 Oct 7;10:1038867. doi: 10.3389/fcell.2022.1038867 (PMC9585323; doi:10.3389/fcell.2022.1038867)
Supplement: Supplementary file 5 [file Presentation1.pdf]

Supplementary material

Supplementary Figure S1: Generation of the voltage sensor hiPSC lines by insertion of the VSFP into the *AAVS1* locus using CRISPR/Cas9

Supplementary Figure S2: Characterization and optical AP recordings of AAVS1-VSFP-hiPSC-vCMs

Supplementary Figure S3: Characterization and optical AP recordings of AAVS1-VSFP-hiPSC-aCMs and -nCMs

Supplementary Table 1: Sequences of primers used for cloning and sequencing

Supplementary Table 2: Primary and secondary antibodies used for immunostaining (IF) and flow cytometry (FC) analysis

Supplementary Video 1a: Movie of d60 homozygous AAVS1-VSFP-hiPSC-vCMs (GFP)

Supplementary Video 1b: Movie of d60 homozygous AAVS1-VSFP-hiPSC-vCMs (RFP)

Supplementary Video 2a: Movie of AAVS1-VSFP-hiPSC-vCMs 3D heart patches at day 21 after reseeding (GFP)

Supplementary Video 2b: Movie of AAVS1-VSFP-hiPSC-vCMs 3D heart patches at day 21 after reseeding (RFP)

Supplementary figure legends

Supplementary Figure S1: Generation of the voltage sensor hiPSC lines by insertion of the VSFP into the *AAVS1* locus using CRISPR/Cas9

(A) A schematic illustrating the mechanism of VSFP function. The membrane depolarization induces structural changes of the voltage-sensing transmembrane domain, leading to closer proximity of the green and red fluorescent proteins, increasing the Förster resonance energy transfer (FRET). **(B)** A schematic depicting the strategy of the CAG-VSFP knock-in into the *AAVS1* locus. Cas9 is targeted by the gRNA (blue) to the *AAVS1* locus in the first intron of the *PPP1R12C* gene and induces a double-stranded break (DSB) 3 bp upstream of the protospacer adjacent motif (PAM, red). Homology-directed repair mediates insertion of the AAVS1-VSFP donor construct into the DSB region. Ex: exon; HA-L/R: left/right homology arms; SA: splice acceptor; T2A: *Thosea asigna* virus 2A peptide; pA: polyadenylation site; pCAG: CAG promoter; puro: puromycin selection marker. P1 (forward primer binding to the first intron of the *PPP1R12C* gene), P2 (reverse primer binding to the right homology arm), P3 (reverse primer binding to the SA-T2A region in the donor pAAVS1-p-CAG-VSFP-polyA construct) indicate primers used for PCR screening of the AAVS1-VSFP-hiPSC clones. **(C)** A schematic showing the screening strategy. Following nucleofection with Cas9-gRNA and donor constructs, the targeted hiPSCs were selected with puromycin and reseeded on 10 cm dishes at the density of 1000 cells per dish. When colonies were big enough, they were cut into two halves, one half was used for cultivation and the other half for sequencing. **(D)** PCR genotyping results of the 24 AAVS1-VSFP-hiPSC clones by amplification of the targeted and non-targeted alleles using three primers, P1, P2, and P3. Homozygous clones were identified by the presence of 1.2 kb amplicon (P1+P3), heterozygous clones by both 1.2 kb (P1+P3) and 1.4 kb (P1+P2) products, and clones without VSFP-cassette insertion by the presence of 1.4 kb (P1+P2) PCR product. Homo: homozygous; het: heterozygous. (**E**) Number of homo-, heterozygous and no-insertion genotypes within analyzed AAVS1-VSFP-hiPSC clones and evaluation of the corresponding editing efficiency. Ins.: insertion; eff.: efficiency. **(F)** Live flow cytometry analysis of GFP and RFP expression in homo- (homo, marked as dark green and dark red, respectively) and heterozygous (het, marked as light green and light red, respectively) AAVS1-VSFP-hiPSC lines. NC, negative control (gray).

Supplementary Figure S2: Characterization and optical AP recordings of AAVS1-VSFP-hiPSC-vCMs

(A) Representative flow cytometry dot plots showing d15 (day 15) homozygous and heterozygous AAVS1-VSFP-hiPSC-vCMs immunostained with antibodies against cTNT and GFP. **(B)** Live flow cytometry analysis of GFP expression in homozygous AAVS1-VSFP-

hiPSCs and AAVS1-VSFP-hiPSC-vCMs at d15, d30, and d60. NC, negative control (gray). **(C)** Live images of GFP and RFP expression in d60 homo- and heterozygous AAVS1-VSFP-hiPSC-vCMs. The white dotted rectangle highlights accumulation of GFP and RFP signal at the cell-cell contacts. **(D)** Spontaneous optical AP measurement of d60 heterozygous AAVS1-VSFP-hiPSC-vCMs. White dotted lines represent region of interest (ROI) used to quantify the GPF and RFP fluorescence signal (left panel). Background-corrected GFP and RFP fluorescence signals derived from the ROI (middle panel). The APs are calculated by RFP/GFP ratio (right panel).

Supplementary Figure S3: Characterization and optical AP recordings of AAVS1-VSFP-hiPSC-aCMs and -nCMs

(A) A representative flow cytometry plot showing d15 (day 15) homozygous AAVS1-VSFP-hiPSC-aCMs immunostained with antibodies against cTNT and GFP. **(B)** A representative image of d60 heterozygous AAVS1-VSFP-hiPSC-aCMs immunostained with antibodies against MLC2v (green) and MLC2a (red). Nuclei were labeled with DAPI (blue). **(C)** Live images of GFP and RFP expression in d60 homozygous AAVS1-VSFP-hiPSC-aCMs. **(D)** Live cytometry analysis of GFP and RFP expression in d60 homozygous AAVS1-VSFP-hiPSC-aCMs. d60 aCMs derived from a control hiPSC line were used as a negative control (NC, gray). **(E)** Quantification of APD90 and APD50 in both homo- and heterozygous AAVS1-VSFP-hiPSC-aCMs at day 60 at spontaneous beating. Data are mean ± SD; N = 434 homozygous and N = 266 heterozygous AAVS1-VSFP-hiPSC-aCMs from n = 5 differentiations per line; *p < 0.0001 (Kruskal-Wallis test). **(F)** Representative flow cytometry plots showing d15 and d60 homozygous AAVS1-VSFP-hiPSC-nCMs immunostained with antibodies against cTNT and GFP. **(G)** Percentage of SHOX2+ and SHOX2- cells in d60 homozygous AAVS1-VSFP-hiPSC-nCMs as determined by the immunostaining for cTNT and SHOX2. Data are mean ± SD; N = 606 nCMs from n = 3 differentiations. **(H)** Live images of GFP and RFP expression in d60 homozygous AAVS1-VSFP-hiPSC-nCMs. **(I)** Live cytometry analysis of GFP and RFP expression in d60 homozygous AAVS1-VSFP-hiPSC-nCMs. d60 nCMs derived from a control hiPSC line were used as a negative control (NC, gray).

Supplementary tables

Supplementary Table 1: Sequences of primers used for cloning and sequencing

**Name**	**Target**	**Sequence (5' to 3')**	
Cloning primer for CAG-VSFP	pcDNA3.1/ Puro-CAG-VSFP-CR plasmid	PacI-polyA Fw	GCATTTAATTAACCAGTGTGGTGGAATTCT GCAGATATC
		SalI-polyA_Rv	CCGGGTCGACCCATAGAGCCCACCGCATC CCCAGCATG
Construct sequencing primers	pAAVS1-p-CAG-VSFP-polyA plasmid	Clover-Fw3	CCTCCAGCTGATTTAGTTGG
		Clover-Fw4	TCGTTGTCACATTCGTCGTC
		Clover-Fw5	AACCGCATCGAGCTGAAGG
		Ruby2-Fw6	CAAGTACCCGAAAGGCATTC
		Ruby2-Fw7	GGATGGACGAGCTGTACAAG
		Ruby2-Fw8	AGTTGATGGTGGTGGCCATC
		pCAG2-Fw11	CTGAGCACGGCCCGGCTTCG
		pCAG2-Fw10	TTCCTTTTATGGCGAGGCGG
		pCAG2-Fw9	AACGCCAATAGGGACTTTCC
		pCAG2-Rv12	CTGCCAAGTAGGAAAGTCCC
AAVS1 locus sequencing before insertion	*AAVS1* locus	AAVS1_Fw1	AGTCCGGACCACTTTGAGCTCTAC
		AAVS1_Rv1	AAGAGCTAGCACAGACTAGAGAG
		AAVS1_Fw2	CATCCTCTTGCTTTCTTTGCCTGG
		AAVS1_Rv2	ACGGAGGAACAATATAAATTGGG
		AAVS1_Fw3	TACACTTCCCAAGAGGAGAAGCAG
		AAVS1_Rv3	CACAGTTGGAGGAGAATCCAC
AAVS1 locus PCR after insertion	*AAVS1* locus after CAG-VSFP insertion	P1_Fw	TCGACTTCCCCTCTTCCGATG
		P2_Rv	CTCAGGTTCTGGGAGAGGGTAG
		P3_Rv	GAGCCTAGGGCCGGGATTCTC

Supplementary Table 2: Primary and secondary antibodies used for immunostaining (IF) and flow cytometry analysis (FC)

**Target**	**Host**	**Reference**	**Concentration**
MLC2v	Rabbit	Proteintech, 10906-1-AP	1:200 (IF)
MLC2a	Mouse	Synaptic systems, 311 011	1:200 (IF)
cTNT	Rabbit	Abcam, ab92546	1:500 (IF/FC)
α-actinin	Mouse	Sigma-Aldrich, A7811	1:250 (IF)
SHOX2	Mouse	Abcam, ab55740	1:400 (IF)
GFP	Chicken	Aves, GFP-1020	1:250 (FC)
IgG control	Rabbit	Abcam, ab37415	1:500 (FC)
IgG control	Chicken	Abcam, ab37382	1:250 (FC)
Anti-rabbit IgG Alexa Fluor 488	Goat	Invitrogen, A11008	1:500 (IF)
Anti-mouse IgG Alexa Fluor 594	Goat	Invitrogen, A11032	1:500 (IF)
Anti-rabbit IgG pacific blue	Goat	Invitrogen, P10994	1:500 (FC)
Anti-chicken IgG Alexa Fluor 647	Donkey	Jackson Immunoresearch, 703-605-155	1:500 (FC)
